# Television viewing time and risk of incident obesity and central obesity: the English longitudinal study of ageing

**DOI:** 10.1186/s40608-015-0042-8

**Published:** 2015-03-01

**Authors:** Lee Smith, Abigail Fisher, Mark Hamer

**Affiliations:** Department of Epidemiology and Public Health, Health Behaviour Research Centre, University College London, England, WC1E 6BT UK; Department of Epidemiology and Public Health, Physical Activity Research Group, University College London, England, WC1E 6BT UK

**Keywords:** Television viewing, Obesity, Older adults

## Abstract

**Background:**

Research suggests television viewing time may be associated with incident obesity and central obesity in young adults. No study has investigated these associations in older English adults.

The aim of this study was to investigate longitudinal associations between television viewing time and incident obesity and central obesity in a sample of older English adults.

Analyses of data from the English Longitudinal Study of Ageing. At baseline (2008), participants reported their television viewing time. Research nurses recorded obesity and central obesity by body mass index and waist circumference, respectively, at four year follow-up. Associations between television viewing time and incident obesity (BMI > 30 kg/m^2^) and central obesity (waist >102 cm men; > 88 cm women) at four year follow-up were examined using adjusted logistic regression. Participants gave full written informed consent to participate in the study and ethical approval was obtained from the London Multicentre Research Ethics Committee.

**Results:**

A total of 3777 initially non-obese participants (aged 64.8 ± 8.6 yrs, 46.4% male) were included in the analyses using BMI as an outcome and 2947 for the analyses using waist circumference. No significant associations were found between television viewing time and incident obesity. A significant association was found between watching ≥6 hrs/d of television (compared to <2 hrs/d) and central obesity (Odds Ratio 1.48; 95% confidence interval 1.07 to 2.03) after adjustment for covariables including physical activity.

**Conclusions:**

In this sample of older community dwelling English adults greater television viewing time was associated with incident central obesity, but not total obesity when measured by BMI. Interventions to reduce the incidence of central obesity in this age group that focus on reducing TV time, as well as targeting other health behaviours (eg, increasing physical activity levels, improving dietary intake) might prove useful.

## Background

In the last 25 years the prevalence of obesity (body mass index [BMI] ≥30 kg/m^2^) in England has more than doubled [1] and in 2010 approximately 26% of English adults (16+ years) were obese [2]. This is of particular concern as prospective studies have found that obesity reduces life expectancy [3,4]. Moreover, obesity is estimated to cost the National Health Service £4.2 billion a year [1].

Television viewing generally involves prolonged periods of sitting and has been found to influence dietary habits by encouraging increased consumption of energy-dense foods [5]. It is therefore reasonable to assume that television viewing time maybe associated with obesity. Two recent systematic reviews summarised the evidence from studies published prior to 2007 that examined the relationship between television viewing time and health outcomes [6,7]. The reviews identified consistent relationships between television viewing time and overweight/obesity. However, identified literature was predominantly of a cross-sectional nature. Therefore, the direction of the observed association cannot be inferred (i.e. does overweight/ obesity cause people to develop more sedentary habits such as television viewing or do individuals become overweight/ obese as a consequence of watching more television). Recently, several prospective studies on television viewing and overweight/ obesity in adult populations have emerged, although mixed results have been reported [8,9]. For example, no relationship between change in participant-reported television viewing time and change in BMI was found over 3 years follow-up in an Australian cohort, although a cross-sectional association was found at baseline, in women only [8]. However, several other studies have reported prospective associations between participant-reported television viewing time and BMI/ obesity in both sexes [9]. In contrast, the Whitehall II prospective study showed that BMI predicted television viewing time at follow-up but the converse was not found [10]. Conflicting findings may be partially explained by the fact that BMI is a poor indicator of adiposity [11], particularly in an elderly sample with onset of sarcopenia. For example, a recent pilot study [12] found associations between objectively measured daily sitting time with levels of liver adiposity and visceral/subcutaneous fat ratio, measured using Magnetic Resonance Imaging (MRI). However, there was no association using BMI, more precise measures of central adiposity in epidemiological studies, such as waist circumference [11], may produce more precise data.

The metabolic risk associated with obesity is closely correlated with a central rather than peripheral fat pattern [13]. Few previous studies investigating the longitudinal association between television viewing time and overweight/obesity have investigated the association between television viewing time and region specific adiposity. In a recent study in a sample of 3846 adults (mean age approximately 48 years) increases in television viewing time over 5 years were associated with increases in waist circumference [14]. Another study found that more frequent television viewing in adolescences and early adulthood was associated with greater BMI gains through to mid-adulthood and with central obesity in mid-life [15]. To our knowledge no study has investigated the longitudinal association between television viewing time and central adiposity in a sample of older adults.

Studying these associations in older adults is important, since adiposity is more likely to be deposited in the abdominal cavity with increasing age [16]. Furthermore, it is thought that BMI becomes a poorer indicator of overall and abdominal adiposity in older people [16,17]. Moreover, the average adult aged >65 years watched more television daily in England than any other age group in 2011 (www.stakeholders.ofcom.org.uk). Studies are needed in older adults (>65 years) to investigate the association between television viewing time and central adiposity, as this age group may be at the greatest risk of central obesity as a consequence of television viewing.

Therefore, the aim of this study was to investigate longitudinal associations between television viewing time and central and total adiposity in a sample of older English adults.

## Methods

The English Longitudinal Study of Ageing (ELSA) is an on-going cohort study containing a nationally representative sample of the English population living in households [18]. The cohort consists of men and women born on or before 29 February 1952. For the purpose of the present analyses data collected during wave 4 (2008 – 2010) were used as the baseline, as this was the first occasion on which information on television viewing was gathered. Clinical information was gathered by nurses in participants’ homes at wave 4 and at follow-up (wave 6; 2012/13). Participants gave full written informed consent to participate in the study and ethical approval was obtained from the London Multicentre Research Ethics Committee.

### Exposure variables: baseline television viewing and physical activity

#### Television viewing time

Participants were asked “how many hours of television do you watch on an ordinary day or evening, that is, Monday to Friday” and “How many hours of television do you normally watch in total over the weekend, that is, Saturday and Sunday.” Average daily time spent watching television was calculated as [(weekday television time x 5) + (Weekend television time)]/7. Next, average daily television was categorised into four categories (<2 hours/day, ≥2 < 4 hrs/d, ≥4 < 6 hrs/d, ≥ 6 hrs/d). This categorisation of average daily television has been used in a previous study [19].

#### Physical activity

Participants were asked how often they took part in vigorous, moderate, and low intensity physical activity. Response options were: more than once a week, once a week, one to three times a month, and hardly ever/ never. Based on response options participants were then categorised into one of three groups (inactive/ moderate at least 1/wk/ vigorous at least 1/wk). For more information on this physical activity measure see Hamer et al. [20]. The physical activity and television viewing measures have been shown to have excellent convergent validity in grading a plethora of psychosocial, physical and biochemical risk factors [20-22].

### Outcome: incidence of obesity

Research nurses measured participants’ body weight using Tanita electronic scales, participants were measured without shoes and in light clothing, and height was measured using a Stadiometer with the Frankfort plane in the horizontal position. BMI was calculated using the standard formulae [weight (kilograms)/height (meters) squared]. Research nurses recorded waist circumference twice mid-way between the iliac crest and lower rib using measuring tape. An average of the first two measurements was used provided these differed by no more than 3 cm; otherwise a third reading was taken and the two closest results utilised. Central obesity was defined as >102 cm in men and >88 cm in women [23].

### Covariates

Age, sex and long standing illness (yes/no) were self-reported. Trained interviewers asked questions on smoking (current, previous, or non-smoker), alcohol intake (daily, at least once a week, monthly, rarely, never), and depressive symptoms (using the eight-item Centre of Epidemiological Studies Depression Scale [24]). Disability was assessed based on participants’ responses to interviewers’ questions on perceived difficulties in six basic activities, such as difficulty dressing, and seven instrumental activities of daily living, such as preparing a hot meal [25]. Participants with difficulties in one or more activities were considered to have some degree of disability. Use of prescribed medication (including medication for diabetes, high blood pressure, cholesterol, blood thinning) was self-reported. These covariates were included in the analyses because they were all hypothesised to be independently associated with both exposures (television viewing time and physical activity) and outcomes (obesity and central obesity).

### Statistical analyses

Characteristics of the study population at baseline were described as means (continuous variables) and percentages (categorical variables). Obese participants (BMI ≥ 30) at baseline were removed from the analysis. We calculated odds ratios (OR) and 95% confidence intervals (CI) for the risk of obesity in relation to television viewing categories (<2 hrs/d, ≥2 < 4 hrs/d, ≥4 < 6 hrs/d, ≥6 hrs/d) using multiple logistic regression. The models were adjusted for age, sex, physical activity, smoking, alcohol, depressive symptoms, long standing illness, disability, cardiovascular (CV) medications. Similar models were run to examine the association with incident central adiposity as the outcome after removing participants with central adiposity at baseline. We tested for statistical interactions with respect to sex although none were found. Thus men and women were pooled together and the analyses were adjusted for sex. All analyses were conducted using SPSS version 21.

## Results

A sample size of 7151 provided complete baseline data although 1884 participants were lost to follow-up, and a further 1490 obese participants at baseline were discarded leaving a final analytic sample of 3777 (aged 64.8 ± 8.6 yrs, 46.4% male) for analyses using BMI as an outcome. For analyses using central obesity as an outcome, 2320 centrally obese participants at baseline were discarded leaving an analytic sample of 2947 participants. Compared with the analytic sample (before removal of baseline obesity), those lost to follow-up were slightly older (64.5 vs. 66.7 yrs, p = 0.001), did not differ in BMI (28.2 vs. 28.4 kg/m^2^, p = 0.10), but had higher baseline television viewing times (5.3 vs. 5.6 hr/d, p = 0.003).

Descriptive characteristics are reported in Table 1. At baseline high levels of television viewing (defined as >6 hr/d) were reported in 24.7% of participants and 14.1% of the sample reported no weekly physical activity of at least moderate intensity. Between baseline and 4 years follow-up, there were 281 and 654 new cases of obesity and central obesity, respectively.

**Table 1:** **Descriptive statistics (baseline analytic sample, n = 3777, after removal of obese [BMI ≥30Kg/m** ^**2**^ **] participants)**

**Variable**	
Age (yrs; mean, SD)	64.8 ± 8.6
Male	46.4
*Physical activity*	
Inactive	14.1
Moderate (at least 1/wk)	48.3
Vigorous (at least 1/wk)	37.5
TV viewing (hr/d; mean, SD)	5.0 ± 4.0
Smoker	12.7
*Alcohol intake*	
Daily	25.7
At least once a week	42.0
Monthly	17.3
Rarely/never	14.9
Long standing illness	46.9
Depressive symptoms (CES-D > 3)	9.9
CV medication	34.7
Disability	17.2
Body mass index (Kg/m^2^; mean, SD)	
Baseline	25.57 ± 2.65
Follow up	25.69 ± 3.02
Waist circumference (cm; mean, SD)^†^	
Baseline	86.93 ± 8.97
Follow up	87.86 ± 20.83

Presented as percentages unless otherwise stated.

^†^Analytic sample excluding centrally obese participants.

In final adjusted models, no significant associations were found between television viewing time and incident of obesity, but physical activity was inversely associated with obesity risk (Table 2). A significant association was found between watching ≥ 6 hrs/d of television (compared to <2 hrs/d) and central obesity, and inverse associations were also observed for physical activity (Table 3).

**Table 2:** **TV viewing, physical activity and incident obesity over 4 years follow-up (N = 3,777)**

**TV/Physical activity exposure**	**Cases/N**	**Model 1**	**Model 2**
		**OR (95% CI)**	**OR (95% CI)**
*TV viewing*			
<2 hrs/d	29/472	1.0 (ref)	1.0 (ref)
≥2 < 4 hrs/d	94/1417	1.08 (0.71, 1.67)	1.02 (0.66, 1.57)
≥4 < 6 hrs/d	71/951	1.23 (0.79, 1.93)	1.08 (0.68, 1.70)
≥6 hrs/d	87/934	1.56 (1.01, 2.42)	1.28 (0.82, 2.01)
*p-trend*		*0.011*	*0.13*
*Physical activity*			
Inactive	68/533	1.0 (ref)	1.0 (ref)
Moderate	127/1824	0.50 (0.36, 0.69)	0.53 (0.38, 0.74)
Vigorous	86/1417	0.43 (0.30, 0.60)	0.48 (0.43, 0.70)
*p-trend*		*<0.001*	*<0.001*

Model 1: adjusted for age and sex.

Model 2: adjusted for age, sex, physical activity (or TV viewing), smoking, alcohol, depressive symptoms, long standing illness, disability (impairment in activities of daily living [ADLs]/ instrumental activities of daily living [IADLs]), CV medications.

Analytic sample excludes obese participants at baseline.

**Table 3:** **TV viewing, physical activity and incident central obesity (men, waist >102 cm; women >88 cm) over 4 years follow-up (N = 2947)**

**TV/Physical activity exposure**	**Cases/N**	**Model 1 OR (95% CI)**	**Model 2 OR (95% CI)**
*TV viewing*			
<2 hrs/d	69/402	1.0 (ref)	1.0 (ref)
≥2 < 4 hrs/d	230/1134	1.20 (0.89, 1.62)	1.19 (0.88, 1.61)
≥4 < 6 hrs/d	162/699	1.40 (1.02, 1.92)	1.25 (0.90, 1.73)
≥6 hrs/d	193/712	1.71 (1.26, 2.34)	1.48 (1.07, 2.03)
*p-trend*		*<0.001*	*0.015*
*Physical activity*			
Inactive	129/405	1.0 (ref)	1.0 (ref)
Moderate	334/1374	0.69 (0.54, 0.88)	0.81 (0.63, 1.06)
Vigorous	191/1168	0.43 (0.33, 0.56)	0.54 (0.40, 0.72)
*p-trend*		*<0.001*	*<0.001*

Model 1: adjusted for age and sex.

Model 2: adjusted for age, sex, physical activity (or TV viewing), smoking, alcohol, depressive symptoms, long standing illness, disability (impairment in activities of daily living [ADLs]/ instrumental activities of daily living [IADLs]), CV medications.

Analytic sample excludes centrally obese participants at baseline.

## Discussion

This is the first study to investigate the longitudinal association between television viewing time and incident obesity and central obesity in a sample of older adults. Participants who reported watching a high level of television (≥6 hrs/d) at baseline were 1.48 times more likely to be centrally obese at four-year follow-up compared to those who reported watching a low level of television, independently of physical activity and other covariates. This finding supports previous literature in younger adults that has investigated the longitudinal association between television viewing time and incident of central obesity. For example, Parsons et al. found that more frequent television viewing in early adulthood is associated with greater central obesity in mid-life [15]. Two plausible pathways which television viewing time may contribute to a higher incident of central obesity include via the act of being sedentary per se and via an increased consumption of energy dense foods [5]. Therefore, television viewing may negatively influence both sides of the energy balance equation. One potential intervention to prevent greater central obesity could be to encourage participants to step during television commercial breaks. Alternatively, interventions may displace TV viewing per se with active outdoor activity.

Interestingly, the present analyses found no significant association between television viewing time and incident of obesity (BMI ≥30), partly consistent with previous literature that has reported mixed results. In the elderly, body fat is more likely to be deposited in the central cavity and thus BMI may be a poor measure of adiposity in this age group [17]. Another reason for reported inconsistencies in the literature is that the associations between television viewing and obesity might be bi-directional [10]. Indeed, we have previously shown that BMI was associated with increased television viewing time over 2 years follow-up in ELSA participants [19]. Consistent with previous literature this study found that higher levels of physical activity are associated with lower central and total obesity [26-28], likely owing to increases in energy expenditure [29].

The prospective design, the large sample of English older adults, and the measure of central obesity are clear strengths of the present analyses. However, the present analyses do have limitations. It was not possible to adjust models for diet, as such data were not collected. Moreover, Self-report television viewing time may suffer from reporting bias, owing to cognitive challenges associated with estimating previous television viewing, and social desirability bias. However, sedentary time questions focusing on television viewing have the strongest reliability and validity among non-occupational sedentary behaviour questions [30]. Some of the clinical variables, such as chronic illness, were also self-reported that may have introduced bias. However, self-reported chronic illness has been previously linked to various objective ageing outcomes in ELSA such as physical function [21]. It should be noted that the findings from the present analyses should not be generalised beyond the demographics (ie, age and ethnicity) of the sample population.

## Conclusions

In this sample of older community dwelling English adults greater television viewing time was associated with incident central obesity, but not total obesity when measured by BMI. Interventions to reduce the incidence of central obesity in this age group that focus on reducing TV time, as well as targeting other health behaviours (eg, increasing physical activity levels, improving dietary intake) might prove useful.

## References

[1] Public Health England. www.noo.org.uk. Accessed 15 Jan 2015.

[2] Hirani V. Adult anthropometric measures, overweight and obesity. Ch.10, Health Survey for England 2010. 2011.

[3] Peeters A, Barendregt JJ, Willekens F (2003). Obesity in adulthood and its consequences for, life expectancy: a life-table analysis. Ann Intern Med.

[4] Fontaine KR, Redden DT, Wang CX, Westfall AO, Allison DB (2003). Years of life lost due to obesity. JAMA.

[5] Lank NH, Vickery CE, Cotugna N, Shade DD (1992). Food commercials during television soap operas: what is the nutrition message?. J Commun Health.

[6] Foster JA, Gore SA, West DS (2006). Altering TV viewing habits: an unexplored strategy for adult obesity intervention?. Am J Health Behav.

[7] Williams D, Raynor H, Ciccolo J (2008). A review of TV viewing and its association with health outcomes in adults. Am J Lifestyle Med.

[8] Crawford DA, Jeffery RW, French SA (1999). Television viewing, physical inactivity and obesity. Int J Obes Relat Metab Disord.

[9] Cournot M, Ruidavets JB, Marquie JC, Esquirol Y, Baracat B, Ferrieres J (2004). Environmental factors associated with body mass index in a population of Southern France. Eur J Prev Cardiol.

[10] Pulsford RM, Stamatakis E, Britton AR, Brunner EJ, Hillsdon MM (2013). Sitting behavior and obesity: evidence from the Whitehall II study. Am J Prev Med.

[11] Burkhauser R, Cawley J (2008). Beyond BMI: the value of more accurate measures of fatness and obesity in social science research. J Health Econ.

[12] Smith L, Thomas EL, Bell JD, Hamer M (2014). The association between objectively measured sitting and standing with body composition: a pilot study using MRI. BMJ Open.

[13] Shen W, Punyanitya M, Chen J (2006). Waist circumference correlates with metabolic syndrome indicators better than percentage fat. Obesity.

[14] Wijndaele K, Healy GN, Dunstan DW (2010). Increased cardiometabolic risk is associated with increased TV viewing time. Med Sci Sports Exerc.

[15] Parsons TJ, Manor O, Power C (2008). Television viewing and obesity: a prospective study in the 1958 British birth cohort. Eur J Clin Nutr.

[16] Seidell JC, Visscher TLS (2000). Body weight and weight change and their health implications for the elderly. Eur J Clin Nutr.

[17] Baumgartner RN, Heymsfield SB, Roche AF (1995). Human-body composition and the epidemiology of chronic disease. Obes Res.

[18] Steptoe A, Breeze E, Banks J, Nazroo J (2013). Chort profile: the English longitudinal study of ageing. Int J Epidemiol.

[19] Gardner B, Lliffe S, Fox K, Jefferis B, Hamer M (2014). Sociodemographic, behavioural and health factors associated with changes in older adults’ TV viewing over 2 years. IJBNPA.

[20] Hamer M, Lavoie KL, Bacon SL (2014). Taking up physical activity in later life and healthy ageing: the English longitudinal study of ageing. Br J Sports Med.

[21] Hamer M, Stamatakis E (2013). Screen-based sedentary behavior, physical activity, and muscle strength in the English longitudinal study of ageing. PLoS One.

[22] Hamer M, Stamatakis E (2013). Prospective study of sedentary behavior, risk of depression, and cognitive impairment. Med Sci Sports Exerc.

[23] International Diabetes Federation. IDF consensus worldwide definitionof the metabolic syndrome. 2006. www.idf.org. Accessed: 15 Jan 2015.

[24] Karim J, Weisz R, Bibi Z, Rehman S. Validation of the eight-item centre for epidemiologic studies depression scale (CES-D) among older adults. Current Psychology. 2014; doi:10.1007/s12144-014-9281-y.

[25] Katz S, Downs TD, Cash HR, Grotz RC (1970). Progress in development of the index of ADL. Gerontologist.

[26] Summerbell CD, Douthwaite W, Whittaker V (2009). The association between diet and physical activity and subsequent excess weight gain and obesity assessed at 5 years of age or older: a systematic review of the epidemiological evidence introduction. Int J Obesity.

[27] Ekelund U, Besson H, Luan JA (2011). Physical activity and gain in abdominal adiposity and body weight: prospective cohort study in 288,498 men and women. Am J Clin Nutr.

[28] Williams PT, Pate RR (2005). Cross-sectional relationships of exercise and age to adiposity in 60,617 male runners. Med Sci Sport Exer.

[29] Kesaniemi YK, Danforth E, Jensen MD, Kopelman PG, Lefebvre P, Reeder BA (2001). Dose–response issues concerning physical activity and health: an evidence-based symposium. Med Sci Sports Exerc.

[30] Clark BK, Sugiyama T, Healy GN, Salmon J, Dunstan DW, Owen N (2009). Validity and reliability of measures of television viewing time and other non-occupational sedentary behaviour of adults: a review. Obes Rev.

